# The Dynamic and Correlation of Skin Temperature and Cardiorespiratory Fitness in Male Endurance Runners

**DOI:** 10.3390/ijerph16162869

**Published:** 2019-08-11

**Authors:** Jonathan Galan-Carracedo, Andrea Suarez-Segade, Myriam Guerra-Balic, Guillermo R. Oviedo

**Affiliations:** 1 FPCEE-Blanquerna, Ramon Llull University, 34 Císter Street, 08022 Barcelona, Spain; 2 Labsportsalud, Laboratory of Exercise Physiology, s/n Verge of Montserrat Street, 08040 Cornella, Spain

**Keywords:** skin temperature, aerobic fitness, endurance

## Abstract

During endurance exercise, skin temperature (Tsk) plays a fundamental role in thermoregulatory processes. Environmental temperature is the biggest determinant of the Tsk. During exercise, the response of the skin temperature might be influenced by aerobic fitness (VO_2peak_). The aim of this study was to analyze and compare the dynamic of Tsk in high (HF) and moderately (MF) fit endurance runners during a progressive maximal stress test. Seventy-nine male endurance runners were classified into HF (*n =* 35; VO_2peak_ = 56.62 ± 4.31 mL/kg/min) and MF (*n =* 44; VO_2peak_ = 47.86 ± 5.29 mL/kg/min) groups. Tsk and cardiovascular data were continuously monitored during an incremental exercise, followed by a recovery period of five minutes. Results revealed that the MF group exhibited lower VO_2peak_, Speed_peak_, ventilation (VE), muscle mass %, and higher BMI and fat mass % than the HF group (all *p* < 0.001). HF had significantly higher Tsk at baseline, and at 60% and 70% of peak workload (all *p* < 0.05). Tsk_peak_ correlated with age, fat mass %, muscle mass %, VO_2peak_, Speed_peak_, HR and VE (all *p* < 0.05). These findings indicate that VO_2peak_ was positively associated with increased Tsk during incremental exercise in male endurance runners.

## 1. Introduction

The ability to thermoregulate is essential for adapting to exercise demands, minimizing changes in body core temperature (Tc) and maintaining physiological homeostasis [1]. Changes in temperature can be perceived at different areas of the human body, where the most critical changes occur for the skin, muscle, and core tissues (i.e., of the rectal, visceral, and esophageal) [2]. Skin temperature (Tsk) plays a fundamental role in body temperature regulation, providing negative and positive auxiliary feedback to the thermoregulatory system. Tsk is the result of the balance between metabolic heat production, heat dissipation to the environment, and tissue temperature [3]. This balance is influenced mainly by the responses of Tc, environment temperature and complex relationships between cutaneous vasodilation and sweating, which facilitate the heat exchange with the environment [4,5]. The delivery of heat from the deeper parts of the body to the skin is accomplished primarily by blood circulation [6], which implies that Tsk can be used as an index to predict thermal changes during exercise [7,8].

During exercise, the impact of environmental conditions influences the skin thermoregulatory response and capacity for heat exchange with the environment [9].

Thermogenesis, from exercise is associated with large hemodynamic changes involving multiple thermoregulatory processes. These changes are reflected in the skin temperature response during exercise [6], and represent a good indication of whether physiological mechanisms are functioning properly, which are vital to the maintenance of thermal homeostasis. 

Thermoregulation is the major process that governs skin blood flow (SkBF) in humans [10] and several studies use SkBF to describe and characterize the skin thermoregulatory response to exercise [6,11]. Several studies had been proposed to estimate the skin thermoregulatory response from SkBF and analyze relationship between Tsk and SkBF during exercise [12]. Kenney and Johnson [13] found that the modification of cutaneous blood flow during exercise depends on the individual level of vasodilation and vasoconstriction. The vasoconstrictor response decreases Tsk, induced by a reduction in SkBF; conversely, the vasodilator response leads to increase Tsk, associated with a substantial increase in SkBF. Accordingly, there is a close link between thermoregulatory vasodilation and increased SkBF (together with sweating), which is essential for heat dissipation during exercise [14].

The relationship of skin thermoregulatory response with Tc, SkBF and environment are modulated by several factors, such as sex [15], an individual’s acclimatization state [16], environmental conditions [17], body composition [18], aging [15], circadian rhythms [19], the wearing of protective clothing [2], hydration status and/or diet [20,21], lifestyle [22], physiological characteristics [23,24], and relevant to this study, physical conditioning [10,25,26]. 

Obviously, the thermogenesis associated with aerobic exercise is a challenge to thermoregulation [10]. In fact, recent studies show that aerobic fitness modifies and improves the thermoregulatory control of SkBF, as manifested by a greater augmentation of skin perfusion for the same increase in core temperature and workload in athletes, in comparison with sedentary subjects [10,11,27,28].

Aerobic fitness level is an important determinant in the health status of individuals of any age. It was reported that the maximum oxygen consumption (VO_2peak_) decreases by about 7% per decade [29]. Based on this discovery, the decrease in skin thermoregulatory capacity, associated with reduced SkBF, may be related to the decline in VO_2peak_ [30]. However, other studies reported that fitness level, associated with regular endurance-type exercise, can induce partial acclimation [16,31] and thereby improve the ability to thermoregulate, enhancing the skin vasodilation response during exercise [11,32]. Boegli [10] concluded that endurance training modifies the skin thermoregulatory response, as manifested by a greater augmentation of skin perfusion and maintenance of active cutaneous vasodilation during exercise [11,28]. Other studies [11,33,34] also reported that physical endurance training and increased VO_2peak_ improve skin thermoregulatory response, which appears to be one of the main elements needed for an effective thermoregulatory active vasodilation response, as well as increased SkBF [30,35]. Moreover, Richmond, Davey, Griggs, and Havenith (2014) [36], found that there was an association between enhanced SkBF response and increased VO_2peak_ after exercise training in older subjects. These changes in VO_2peak_ seemed to affect Tsk responses. Périard et al. (2001) [16] also postulated that a better skin thermoregulatory response during endurance exercise could indicate a higher cardiorespiratory fitness level, despite aging.

Despite these discoveries, Tsk response remains unstudied as an independent parameter in the control of body temperature during exercise [37]. Little attention has been paid to Tsk response, resulting in a key factor in providing better insight into the behavior of the thermoregulatory system during exercise. In this sense, it is unclear if the level of aerobic fitness can influence Tsk response, irrespective of age [38,39]. To the best of our knowledge, the dynamic of Tsk during a maximal stress test and its association with cardiorespiratory fitness in male endurance runners has not been directly studied. Therefore, the main objective of the present study was to analyze and compare the dynamic of Tsk during a maximal stress test in both high (HF) and moderately (MF) fit male endurance runners. Secondly, we analyzed the correlations between Tsk and cardiorespiratory variables. It was hypothesized that HF runners have the ability to maintain higher Tsk responses than MF runners during maximal exercise. 

## 2. Materials and Methods 

### 2.1. Study Design and Participants

The present study followed a cross-sectional research design. A total of 79 trained male endurance runners participated in this study from running and triathlon teams. Participants were eligible for the study if they met the following inclusion criteria: all subjects had to be at an aerobic fitness level ≥40th percentile, based on the American College of Sports Medicine (ACSM) age-specific cardiorespiratory fitness classification [40]; regular running training, at least three times/week and a minimum of 120–150 min/week; having competed in an endurance event (>5 km) within 3 months prior to the study; and ≥3 years of competitive running experience [41]. All subjects were nonsmokers, deemed healthy (assessed by completion of a general health questionnaire), with no known cardiovascular or metabolic disorders, and were not taking medication that had the potential to impact cardiovascular or thermoregulatory function. All significant inclusion criteria were the same for all runners, except age, which was 18–50 years.

Participants were divided into two groups: the HF group was >80th percentile and the MF group was ≤80th percentile based on the ACSM age-specific cardiorespiratory fitness classification. The HF (*n =* 35; age 36 ± 8 years) and MF (*n =* 44; age 37 ± 9 years) participants performed a maximal exercise test on a treadmill. Before beginning the study, the participants had the protocol explained to then, as well as the testing procedures and time required for the study. All the participants signed an informed consent form. The procedures of this study followed the Helsinki guidelines [42] for ethical behavior and was approved by the local Human Research Ethics Committee of Blanquerna, University of Ramon Llull.

### 2.2. Experimental Procedure

Because external and internal factors have an influence on the day of the test, in order to measure Tsk under similar conditions, the following characteristics were used as exclusion criteria and participants were asked not to: (a) smoke or drink alcohol at least 12 h before the test; (b) sunbathe or be exposed to UV rays; (c) use body lotions and creams; (d) carry out high-intensity or exhaustive exercise < 24 h before the test; (e) eat at least 2 h before the test and refrain from having a heavy meal; (f) drink coffee or stimulants 2 h before the test; and (g) use medications, such as antipyretics or diuretics, or any dietary supplement that could potentially interfere with water homeostasis and body temperature in the previous two weeks. Every participant was measured at a similar time in order to reduce the intra-subject effect of the circadian cycle. Finally, heat acclimation can influence the overall control of Tsk during exercise [43]. Consequently, we decided to perform our study in winter and spring, avoiding the possible effects of heat acclimation during the warmer season.

### 2.3. Anthropometric Measurements

Body mass was measured to the nearest 0.1 kg on a digital scale (Seca 861, Hamburg, Germany), with the subject wearing lightweight clothing and no shoes. Body height was measured using a stadiometer to the nearest 0.1 cm (Seca 225, Seca, Hamburg, Germany). Body mass index (BMI) (kg/m^2^) was calculated using body mass and body height, following the recommendations of the International Society for the Advancement of Kinanthropometry [44].

Body density was estimated using the seven site skinfold equation (chest, axilla, subscapular, midaxillary, triceps, abdominal and thigh) developed by Jackson and Pollock [45]. Skinfold measurements were taken on the right side of the body three times by the same researcher using a Holtain skinfold caliper (Holtain Ltd., Walles, UK) and following the ISAK guidelines [44]. Body fat percentage (%) was calculated using the Siri equation [46], with muscle mass percentage determined thereafter. Muscle mass percentage (%) was determined together with bone and organs percentages using the equation of the sum of seven perimeters (arm, contracted arm, forearm, wrist, chest, upper thigh, medial thigh and calf) and 6 diameters (biacromial iliac spine, breadth, chest, humerus, femur, anterior-posterior thoracic and transverse thoracic) [47]. Finally, all tests were performed in the morning between 9 am to 12 pm to reduce the intra-subject effect of the circadian cycle. The test was carried out in a controlled environment, where conditions were maintained at 22 ± 1 °C and 50 ± 5% relative humidity.

### 2.4. Cardiorespiratory Fitness Assessment

Each participant performed an incremental test on a treadmill (Quasar model, HP Cosmos sports and medical gmbh, Nussdorf-Traunstein, Germany). During the test, Tsk (using Biopac Student Lab software) was monitored, as were cardio-vascular and ventilation responses using a gas analyzer. Oxygen consumption (VO_2_), CO_2_ production (VCO_2_), ventilation (VE) and the respiratory exchange ratio (RER = VCO_2_/VO_2_) were measured breath-by-breath with an automatic gas analysis system (Ergospirometer, Powercube-Ergo, Gansborn Medizine Electronic GmbH, Niederlaur, Germany). During the test, participants started at a speed of 7 km/h, which increased by 1 km/h every 2 min until exhaustion. The participants performed the test at a constant slope (1.5%). All cardiorespiratory variables, rate of perceived exertion (RPE) and Tsk were monitored at rest, during exercise and during the 5 min recovery period. The twelve lead electrocardiograms (CardioScan v.4.0, DM Software, Staline, NV, USA) and heart rate (HR) (Polar RS800CX, Polar Electro, Lake Success, NY, USA) were monitored continuously during the test and five minutes of the recovery period. Perception of fatigue was reported by means of a 6–20 point Borg scale [48].

### 2.5. Skin Temperature Assessment

On the day of test, subjects reported normal hydration. Skin temperature was continuously recorded by a Tsk sensor (thermistor sensor, TSD202D, Biopac Systems Inc., Goleta, CA, USA) that was placed on the left pectoralis muscle 2.5 cm medial and 2.5 cm above the nipple. Accuracy and precision of the device model is ± 0.2 °C. Before monitoring Tsk, participants were acclimated to the environment by standing in the room for 15 min. During the incremental test, Tsk data was recorded every half second and the mean value of each ten seconds was used for data analysis (Biopac Student Lab Analysis software, Biopac Systems Inc., Goleta, CA, USA). 

### 2.6. Statistical Analysis 

After checking the normal distribution of the variables (Kolmogorov-Smirnov test), a one-way ANOVA with post-hoc Bonferroni test was used to determine between-group differences. Pearson’s r correlation was used to analyze the associations between Tsk and cardiorespiratory variables.

Finally, a multiple linear regression identifying significant variables was developed with Tsk used as the response variable. The explanatory variables were those found to be significantly correlated (*p* < 0.05) with Tsk, using a linear relationship. Multicollinearity was checked with the variance inflation factor (VIF), which needs to be below 10 for all predictor variables [49].

The critical values for statistical significance were assumed to be at an alpha level of less than 0.05. Statistical analyses were conducted using the Statistical Package for the Social Sciences (IBM SPSS, v 22.0, Chicago, IL, USA).

## 3. Results

### 3.1. Participant Characteristics and Cardiorespiratory Assessments

Table 1 shows general and physiological characteristics of the participants. All subjects were similar in height and age. Body weight and BMI were significantly higher in the MF group (*p* = 0.001), compared with the subjects of the HF group. The HF group had a higher muscle mass % and a lower fat mass % than the MF group (all *p* < 0.001).

#### Cardiovascular Response

VO_2peak_ was significantly higher in the HF group than the MF group (56.62 ± 4.31 vs. 47.86 ± 5.29 mL/kg/min, *p* < 0.05). Also, both the maximal speed reached during the test and VE were higher in the HF group compared with the MF group (all *p* < 0.001). Significant differences in peak HR (HR_peak_) and peak RER (RER_peak_) were not found.

### 3.2. Skin Temperature Measurements

Results from the analysis of Tsk are shown in Table 2. These results provide the mean Tsk responses at rest (baseline), during exercise, and after exercise during the recovery period. Baseline Tsk was significantly higher (*p* = 0.049) in the HF group compared with the MF group.

**Figure 1:**
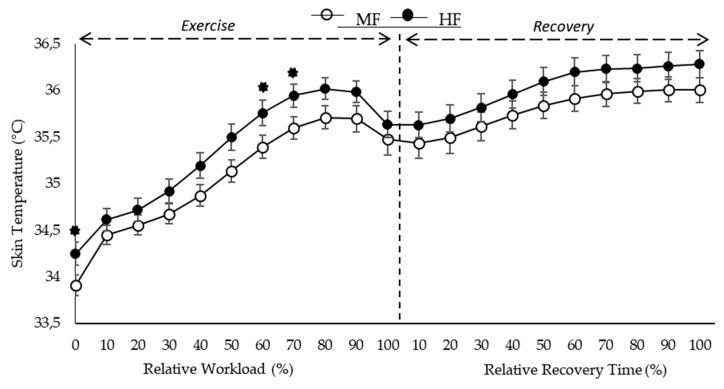
Values are means ± SEM. The black circles represent the highly fit endurance runners (HF) group and the white circles represent the moderately fit endurance runners (MF) group. Significant level was set at *p* < 0.05. Analysis was performed at the end of each relative workload of exercise and recovery period. * Significant differences between highly and moderately fit endurance runners.

Values of Tsk at each percentage of peak workload during the incremental test are shown in Figure 1. Mean Tsk and standard error (SEM) of both groups during exercise are shown in Figure 1. This figure also shows mean Tsk and SEM for each group during the recovery period. During the test, Tsk values were higher in the HF group compared with the MF group (Figure 1). However, these were not statistically significant differences. As the duration and intensity of the test increased, the maximal Tsk (Tsk_peak_) reached was lower in the MF group compared with the HF group throughout exercise (35.90 ± 0.79 vs. 36.20 ± 0.60 °C, respectively). Nevertheless, there were not statistically significant differences between groups (Figure 1). Similarly, during the recovery period, the peak values of Tsk were higher in the HF group compared with the MF group (Figure 1). Both groups reached their Tsk_peak_ during recovery. However, there were not statistically significant differences between groups.

During the test, irrespective of Tsk_peak_ and fitness level, the increase of Tsk from Tsk_baseline_ to Tsk_peak_ was not statistically different (1.96 ± 0.65 vs. 1.99 ± 0.92 °C in HF and MF, respectively) during the test. The difference between the Tsk at the beginning of the recovery period and the higher Tsk achieved in this period for both groups was not statistically different (0.65 ± 0.56 vs. 0.54 ± 0.75 °C in HF and MF, respectively) (Figure 1).

Figure 1 also illustrates the mean responses of Tsk for each group during the recovery period, in which there were not significant differences between groups. The comparison of percentage workload (%) of Tsk responses in the HF and MF subjects during the test and recovery period are shown in Figure 1. Throughout the exercise period, the Tsk response for the HF group was significantly higher at baseline (*p* = 0.049), at 60% (*p* = 0.048) and 70% (*p* = 0.048) of peak workload (%) compared with the MF group (Figure 1). There were no other differences in Tsk among the groups during test and recovery periods (Figure 1). The mean values of Tsk at the end of the test were slightly higher in the HF group, nevertheless, the difference among groups was not significant (HF 35.70 ± 0.77 vs. MF 35.50 ± 1.07 °C). During the incremental test both groups reached stable Tsk values (plateau) at 80 to 90% of peak workload (Figure 1). After reaching the plateau, Tsk started decreasing in both groups (HF = 0.50 ± 0.50 vs. LF = 0.41 ± 0.49 °C). The Tsk_peak_ during the recovery period was greater than the Tsk_peak_ reached during exercise in both groups (HF 36.38 ± 0.79 °C vs. MF 36.14 ± 0.81 °C, respectively), and was also greater than the final Tsk value after recovery.

As showed in Table 3, Tsk_peak_ of both group was inversely correlated with fat mass %. On the other hand, Tsk was positively correlated with age, muscle mass %, VO_2peak_, HR_peak_, VE_peak_ (*p* < 0.05) and Speed_peak_ (*p* = 0.002) (Table 3).

The multivariate linear regression that was used to identify factors that significantly affected Tsk_peak_ showed that Speed_peak_ had a significant effect on Tsk_peak_. Although HR_peak_ has positive effects on Tsk_peak_, no significant difference was found between groups for this variable (Table 4).

## 4. Discussion

In this study we hypothesized that higher aerobic capacity could be associated with an enhanced response of Tsk in male endurance runners. This hypothesis was tested by comparing two groups of male endurance runners with different levels of aerobic capacity during a maximal exercise test. We observed that the HF group achieve higher Tsk values compared with the MF group, however, the Tsk_peak_ achieved by the groups were not statistically different. As showed in Figure 1, the Tsk dynamic in both groups followed a similar pattern. This may be due to the fact that all subjects were in good physical condition, based on their VO_2peak_ [50]. It is unclear if the observed larger increases in Tsk for the HF group could be due to the fact that subjects with higher aerobic fitness levels may have a better skin thermoregulatory response during exercise. 

The Tsk dynamic during the incremental test can be divided into three parts: (i) initial rise to Tsk_peak_, at around 80% of workload, with an increase in Tsk in the HF and MF groups of 1.96 °C and 1.99 °C, respectively; (ii) a plateau of Tsk at 80% to 90% of peak workload in both groups and, (iii) a decrease in Tsk until the end of exercise. Accordingly, this pattern of increases in Tsk during exercise is consistent with previous observations in individuals with high fitness levels [10]. Our results show that as exercise intensity and VO_2_ increase during the test, Tsk increases until 80–90% of peak workload is reached. It is likely that this continuous increase in Tsk is associated with active cutaneous vasodilation, resulting from increased absolute SkBF as exercise intensity increases [25]. At 80% to 90% of peak workload (Figure 1) we observed a plateau of the Tsk on both groups [5]. From this point, both groups showed a critical and sudden decrease in Tsk, which may likely be associated with decreased SkBF, cutaneous vasodilation and the formation of sweating that will dissipate body heat. Based on previous studies [8], a Tsk plateau at high exercise intensities shows a withdrawal of active vasodilation, which reduces the ability of the athlete to dissipate heat, causing cutaneous vasoconstriction, which is associated with increased adrenergic activity [51]. Our results also seem to confirm the results found by Zontak et al. (1998) [6], in which the rate of decrement of the Tsk was dependent on the intensity of the workload.

During the initial period of the recovery phase, Tsk values increased quickly in both groups due to the need to dissipate heat after exercise. In this phase, our objective was to analyze and compare the behavior and increases in Tsk in both groups. Another study also found similar patterns, and demonstrated that these increases in Tsk reflect the convective transfer of heat from the core to the periphery [37].

Skin temperature is influenced by fitness level, as well as by other variables such as body composition, fat and muscle mass %, age, and other cardiovascular variables, like VE and HR. Aerobic fitness level, along with age, appear to be the most important limiting factors in the cutaneous vasodilation response to exercise [23]. Therefore, physical inactivity and aging contribute to reducing VO_2peak_, which decreases heat dissipation capacity and, consequently, body temperature control during strenuous exercise [52,53]. The MF subjects had a higher BMI, fat mass %, and lower muscle mass and VO_2peak_ (Table 1). These variables and age itself have all been found independently and negatively associated with Tsk_peak_, which is related to maximal cutaneous vasodilation [10].

The result of the multiple linear regression shows that Speed_peak_ has a significant effect on the Tsk_peak_ of the participants. Nevertheless, these results should be interpreted with caution as only 15% of the variance of the Tsk_peak_ is explained by the model.

The results of our study are similar to the results of Merla et al. (2005) [4], in which changes in Tsk during exercise were associated with highly trained individuals, due to a major activation of the sympathetic active vasodilator system and better thermal adaptations [54]. These findings may be linked to direct and substantial vasodilation of blood vessels [55], which contributes importantly to SkBF, helping to reach high Tsk values. Stapleton et al. [30] also found that higher levels of aerobic fitness were associated with an increased rate of Tsk for a given increase in mean body temperature and exercise intensity. 

We conclude that despite between-group differences in fitness level, our results did not show significant differences in Tsk increases between both groups. However, we provide evidence that a moderate to high fitness level may enhance skin thermoregulation response, at least in the torso where the skin temperature sensor was located. This influence of aerobic capacity shows a close relationship between Tsk and cardiovascular responses to relative exercise intensity.

Based on previous studies [56,57] and in our findings, it is clear that the response of the Tsk plays an important role in the thermoregulatory process during maximal exercise. Despite that Tsk is an important measure and contributor for the thermoregulation and aerobic performance, it should be taken into account that Tsk is mainly a consequence of other important factors such as Tc, SkBF and environmental temperature [12]. 

It is important to acknowledge the limitations of this study. Firstly, between-group difference on Tsk_peak_, which was ~0.3 °C, should be interpreted with caution as the accuracy of the device assessing the Tsk is ±0.2 °C. Nevertheless, all participants were assessed by using the same device and methodology, so all assessments may present the same measurement error. Secondly, this is a cross-sectional study and did not study the effects of specific endurance training programs, where the differences between both groups may be due to different physiological capacity to control the skin thermoregulatory response during exercise. Thirdly, Tc was not measured during the incremental test. Core temperature could show that differences in body temperature are due to differences in fitness level, as reported by the literature [5,30,51]. The relationship of this thermal behavior with the Tc and other metabolic responses could help to better determine thermoregulatory response during exercise. Hence, it would be of interest to study the relationship of skin thermal behavior related to Tc and other metabolic responses during graded and progressive exercise in different populations. Finally, sweat on the skin surface at the end of exercise could have influenced Tsk data and should also be considered a limitation of the present study. Ammer (2009) [58] suggested that a film of water on the skin may work as a filter and, therefore, could lead to an underestimation of thermal data. 

The runners who participated in this study presented a different range of aerobic fitness and practiced different endurance sports activities, such as distance running, mountain running and triathlon. This is a strength when it comes to ensuring that the results of the present study not only apply to runners with high aerobic fitness, but also to those exercising at a high recreational level. Future studies should study the skin thermoregulatory response in female endurance runners, and compare the results with those of their male counterparts. Also, it should be interesting to compare and analyze the difference between peripheral and core temperature of male and female runners. 

## 5. Conclusions

The main findings of the present study are that VO_2peak_ is positively associated with Tsk_peak_ rates. However, our results do not show that a higher level of aerobic fitness in male endurance runners contributes to achieve higher Tsk, or a greater thermoregulatory or physiological capacity to dissipate heat during exercise. These observations are of considerable interest in view of our results, which also found that Speed_peak_ is a primary contributor to the increased Tsk response. The dynamic response of Tsk to exercise reflects the balance of hemodynamic and thermoregulatory processes, and may serve as a tool for assessing the integrity of these mechanisms as part of the circulatory system, which interacts with the thermal and hemodynamic responses. These findings might able to contribute to enhancing performance in various endurance or even team sports, where the relationship between aerobic endurance and thermal control can limit endurance performance. More research and future studies are required to determine how the thermoregulatory control of Tsk could substantially affect aerobic performance.

## Figures and Tables

**Table 1:** General characteristics of whole sample and of the two study groups.

Variables	*n =* 79	HF (*n =* 35)	MF (*n =* 44)	*p*-Value
Age (years)	36 *±* 9	36 *±* 8	37 *±* 9	0.734
Height (cm)	177 *±* 0.1	176 *±* 0.5	178 *±* 0.6	0.189
Weight (kg)	75.62 *±* 7.56	72.72 *±* 5.67	77.93 *±* 8.12	0.001
BMI (kg/m^2^)	24.03 *±* 2.11	23.23 *±* 1.50	24.68 *±* 2.32	0.001
Fat mass (%)	14.04 *±* 4.52	11.69 *±* 3.2	15.91 *±* 4.59	<0.001
Muscle mass (%)	45 *±* 3.48	46.51 *±* 2.70	43.80 *±* 3.61	<0.001
VO_2peak_ (L/min)	3.82 *±* 0.38	4.02 *±* 0.35	3.65 *±* 0.33	0.001
VO_2peak_ (mL/kg/min)	51.74 *±* 6.54	56.62 *±* 4.31	47.86 *±* 5.29	<0.001
HR (beat/min)	181 *±* 9	182 *±* 8	181 *±* 11	0.554
RER (VCO_2_/VO_2_)	1.05 *±* 0.05	1.05 *±* 0.05	1.04 *±* 0.05	0.590
VE (L/m)	136 *±* 20	145 *±* 21	129 *±* 17	<0.001
Speed_peak_ (km/h)	15.91 *±* 1.78	16.98 *±* 1.50	15.06 *±* 1.50	<0.001
Time spent (sec)	1200 *±* 212	1329 *±* 181	1099 *±* 180	<0.001

Values are means ± SD. Abbreviations: BMI (body mass index), VO_2peak_ (peak oxygen consumption), HR (heart rate), RER (respiratory exchange ratio), VE (ventilation), Speed_peak_ (peak speed), HF (highly fit endurance runners), MF (moderately fit endurance runners).

**Table 2:** Skin temperature response in an incremental exercise test until volitional exhaustion in high and moderately fit endurance runners.

Variables	*n =* 79	HF (*n =* 35)	MF (*n =* 44)	*p*-Value
Tsk_baseline_ (°C)	34.06 ± 0.75	34.24 ± 0.84	33.91 ± 0.74	0.049
Tsk_peak_ (°C)	36.03 ± 0.73	36.20 ± 0.60	35.90 ± 0.79	0.062
Tsk_final_ (°C)	35.59 ± 0.95	35.70 ± 0.77	35.50 ± 1.07	0.322
Tsk variation from Tsk_baseline_ to Tsk_peak_ (°C)	1.97 ± 0.81	1.96 ± 0.65	1.99 ± 0.92	0.833
Tsk variation from Tsk_baseline_ to Tsk_final_ (°C)	1.53 ± 1.03	1.49 ± 0.84	1.56 ± 1.16	0.747
Tsk variation from Tsk_peak_ to Tsk_final_ (°C)	−0.45 ± 0.49	−0.50 ± 0.50	−0.41 ± 0.49	0.413
Time-duration from Tsk_baseline_ to Tsk_peak_ (s)	996 ± 240	1105 ± 244	910 ± 201	<0.001
Time-duration from Tsk_peak_ to Tsk_final_ (s)	204 ± 163	223 ± 159	189 ± 167	0.348
Tsk_peak_ during recovery (°C)	36.25 ± 0.81	36.38 ± 0.79	36.14 ± 0.81	0.187
Tsk_final_ during recovery (°C)	36.12 ± 0.88	36.28 ± 0.87	36 ± 0.88	0.167
Tsk variation from Tsk_final_ at end of exercise to Tsk_peak_ during recovery (°C)	0.71 ± 0.59	0.76 ± 0.56	0.68 ± 0.61	0.557
Tsk variation from Tsk_final_ at end of exercise to Tsk_final_ at end of recovery (°C)	0.60 ± 0.68	0.65 ± 0.56	0.54 ± 0.75	0.452

Values are means ± SD. Abbreviations: Tsk (skin temperature), Tskbaseline (baseline skin temperature), Tskpeak (maximal skin temperature), Tskfinal (skin temperature at the end of exercise/recovery), HF (highly fit endurance runners), MF (moderately fit endurance runners).

**Table 3:** Correlation Coefficients and *p*-values among Tsk_peak_ and Age, BMI, Fat mass %, Muscle mass %, VO_2peak_, HR_peak_, RER_peak_, VE_peak_ and Speed_peak_.

Variables	Correlation Coefficient	*p*-Value
Age, years	0.306	0.006
BMI (kg/m^2^)	−147	0.196
Fat mass (%)	−276	0.014
Muscle mass (%)	0.263	0.019
VO_2peak_ (ml/kg/min)	0.299	0.007
HR_peak_ (beat/min)	0.286	0.011
RER_peak_	−035	0.760
VE_peak_ (L/min)	0.256	0.023
Speed_peak_ (km/h)	0.337	0.002

Values are means ± SD. Abbreviations: Tsk_peak_ (peak skin temperature), BMI (body mass index), VO_2peak_ (peak oxygen consumption), HR_peak_ (peak heart rate), RER_peak_ (peak respiratory exchange ratio), VE_peak_ (peak ventilation) and Speed_peak_ (peak speed).

**Table 4:** Variables that affect Peak Skin Temperature (Tsk_peak_).

Variables	β	β Error	*T*	*p*	VIF
(Constant)	31.668	1.453	21.790	<0.001	-
Speed_peak_	0.111	0.046	2.392	0.019	1.139
HR_peak_	0.014	0.008	1.694	0.094	1.138

Regression model statistically significant *p* = 0.002; *R^2^* = 0.146. Abbreviations: Tsk_peak_ (peak skin temperature), VIF (variance inflation factor); Speed_peak_ (peak speed) and HR_peak_ (peak heart rate).
